# Columnar cell lesions and subsequent breast cancer risk: a nested case-control study

**DOI:** 10.1186/bcr2624

**Published:** 2010-08-06

**Authors:** Sarah A Aroner, Laura C Collins, Stuart J Schnitt, James L Connolly, Graham A Colditz, Rulla M Tamimi

**Affiliations:** 1 Department of Epidemiology, Harvard School of Public Health, 677 Huntington Avenue, Boston, MA 02115, USA; 2 Department of Pathology, Beth Israel Deaconess Medical Center and Harvard Medical School, 330 Brookline Avenue, Boston, MA 02115, USA; 3 Department of Surgery, Washington University School of Medicine, 660 S. Euclid Avenue, St. Louis, MO 63110, USA; 4 Channing Laboratory, Department of Medicine, Brigham and Women's Hospital and Harvard Medical School, 181 Longwood Avenue, Boston, MA 02115, USA

## Abstract

**Introduction:**

Histologic and genetic evidence suggests that at least some columnar cell lesions (CCL) of the breast represent precursor lesions in the low-grade breast neoplasia pathway. However, the risk of subsequent breast cancer associated with the presence of CCL in a benign breast biopsy is poorly understood.

**Methods:**

The authors examined the association between the presence of CCL and subsequent breast cancer risk in a nested case-control study of benign breast disease (BBD) and breast cancer within the Nurses' Health Studies (394 cases, 1,606 controls). Benign breast biopsy slides were reviewed by pathologists and CCL presence assessed. Logistic regression was used to compute odds ratios (ORs) and 95% confidence intervals (CIs) for the association between CCL and breast cancer risk.

**Results:**

Women with CCL (140 cases, 448 controls) had an increased risk of breast cancer compared with those without CCL (OR = 1.44, 95% CI: 1.14 to 1.83), although this was attenuated and became non-significant after adjustment for the histologic category of BBD (OR = 1.20, 95% CI: 0.94 to 1.54). CCL presence was associated with the greatest risk of breast cancer for those with nonproliferative BBD (OR = 1.36, 95% CI: 0.79 to 2.37) and the lowest risk for those with atypical hyperplasia (AH) (OR = 1.10, 95% CI: 0.65 to 1.87); however, this apparent heterogeneity in risk across BBD categories was not significant (*P *for interaction between CCL presence and BBD category = 0.77).

**Conclusions:**

These results provide evidence that CCL may be an important marker of breast cancer risk in women with BBD but suggest that CCL do not increase breast cancer risk independently of concurrent proliferative changes in the breast.

## Introduction

With the widespread adoption of screening mammography in the past several decades, columnar cell lesions (CCL) of the breast have become a frequent finding in breast biopsies. In fact, these lesions have been reported to be present in nearly half of biopsies performed for mammographic microcalcifications and have been identified as the source of three-quarters of biopsy-detected microcalcifications [1]. CCL is a broad designation that encompasses a wide range of histologic changes ranging from alterations of the epithelium with no architectural or cytologic atypia to changes resembling ductal carcinoma *in situ *(DCIS) [2]. Although these lesions have been recognized for some time, the diverse terminology used to describe such lesions has hindered the understanding of their clinical significance. The myriad of names used to describe these lesions include "blunt duct adenosis" [3,4], "clinging carcinoma" [5,6], "columnar alteration with prominent apical snouts and secretions" [1], "atypical ductal cells with apocrine snouts" [7], "atypical cystic lobules" [8-10], and "well-differentiated DCIS with a clinging architecture" [11]. The increasing use of the term "columnar cell lesions" and the standardization of the classification scheme for these lesions [2] is facilitating the study of CCL as a potential breast cancer risk factor.

Some of the earliest evidence suggesting that CCL may be premalignant was their frequent detection near known precancerous and cancerous changes in the breast. Several studies have reported a high frequency of CCL and low-grade DCIS in the same breast [1,8,12], with concurrent CCL and DCIS commonly occurring in the same or adjacent terminal duct lobular units [1]. Co-occurrence of CCL and low-grade invasive carcinomas, particularly tubular carcinoma, has also been observed [7,13-16]. The presence of CCL in proximity to invasive and non-invasive breast cancer suggests that CCL may have the potential to undergo malignant transformation, although CCL may simply be markers for other pre-cancerous conditions in the breast.

Mounting histopathologic and molecular evidence indicates that CCL may indeed be early precursors to low-grade invasive breast carcinomas. CCL have been proposed as breast cancer precursors largely because of the cytologic and architectural similarities of more advanced CCL to atypical hyperplasia (AH), a suspected precancerous abnormality, and DCIS, a well-established precursor to invasive carcinoma [1,8,10]. In many cases, the cellular features of advanced CCL so closely resemble those of low-grade DCIS that the distinction between the two lesions can be challenging [1,8]. Genetic alterations in CCL following a similar progression as the morphological changes have also been observed, and the mutations in the most advanced lesions have been found to be very similar to those in DCIS or invasive cancer [17-19]. Supporting these findings is evidence of an immunophenotypic link between CCL and breast carcinoma, with advanced CCL displaying an immunohistochemical profile similar to that of DCIS and low-grade invasive cancer [8,18,20-22].

Descriptive studies among patients with advanced CCL, although unable to quantify the elevation in breast cancer risk associated with CCL, suggest that CCL are unlikely to be a strong risk factor for subsequent breast cancer. While Martel *et al. *did find some suggestion of an increased risk of breast cancer subsequent to CCL, as 9 out of 63 participants with advanced CCL (14.3%) developed subsequent infiltrating carcinomas after eight years of follow-up [23], most other descriptive studies have found little evidence for an association between CCL and subsequent breast cancer. In fact, De Mascarel *et al*. found that none of 84 patients with advanced CCL alone developed subsequent breast cancer over a 10-year follow-up [24]. Similarly, no cases of invasive breast cancer were found among 59 patients with CCL followed for a median of 5.4 years in the European Organization for Research and Treatment of Cancer randomized clinical trial [11] or among 25 patients with CCL followed for 19.2 years in a retrospective analysis by Eusebi *et al. *[6]. Although these descriptive studies have provided insight into the natural history of BBD, most were limited by their small sample sizes and short follow-up, and their absence of a comparison group without CCL precludes assessment of the risk associated with CCL.

The very limited epidemiologic evidence on CCL suggests that CCL may be markers for concurrent atypical proliferative changes in the breast rather than breast cancer precursors [25-27]. Given the scarcity of epidemiologic data on the nature of these lesions, we undertook a study of CCL in relation to breast cancer risk using data from an established case-control study of benign breast disease (BBD) within the Nurses' Health Study (NHS) and the Nurses' Health Study II.

## Materials and methods

### Study population

The NHS is an ongoing prospective cohort study that began in 1976, when 121,700 female registered nurses between ages 30 and 55 years completed a mailed, self-administered questionnaire regarding their health behaviors, lifestyle factors, and medical histories. Follow-up questionnaires have been sent to participants every two years to obtain updated information. The biennial questionnaires have assessed a variety of known and suspected risk factors for breast cancer, including history of BBD. On the 1976, 1978, and 1980 questionnaires, participants were asked if they had ever been diagnosed with fibrocystic or other BBD and whether this diagnosis had required hospitalization; from 1982 onward, the questionnaires have inquired specifically about BBD confirmed by biopsy. Deaths are reported by family members and the postal service, and regular searches of the computerized National Death Index are also conducted [28]. The NHS II is a separate cohort study consisting of 116,609 female registered nurses who were between the ages of 25 and 42 years when the study began in 1989. The follow-up methods used in this cohort are very similar to those for the original NHS. On each biennial questionnaire, participants have been asked if they have ever been diagnosed with BBD and, if yes, whether the diagnosis was confirmed by biopsy or aspiration. The follow-up rate for each two-year cycle has been greater than 90% of the original cohorts.

We conducted a nested case-control study to assess morphological changes in benign breast lesions and breast cancer risk among participants in the NHS and NHS II who had reported a previous diagnosis of BBD that was confirmed by biopsy. Within this subcohort, eligible cases were women who reported a first diagnosis of breast cancer between 1976 and the return of the 1996 questionnaire in the NHS or between 1989 and return of the 1995 questionnaire in the NHS II. Self-reported breast cancers were confirmed by review of medical records, and both invasive breast cancer and carcinoma *in situ *were included in the study. Eligible controls were women who completed the questionnaire for the same year that the breast cancer case was reported and had a previous diagnosis of biopsy-confirmed BBD, but were free from breast cancer; within each NHS cohort, they were matched to breast cancer cases on year of birth and year of diagnosis of BBD. We attempted to identify four matched controls for each case, but this was not always possible for logistical reasons. Informed consent was obtained from all participants. The study was approved by the Human Research Committee of Brigham and Women's Hospital, Boston, Massachusetts.

### Benign breast biopsy specimens

Eligible cases and controls were contacted for permission to obtain their BBD pathology records and biopsy specimens, and specimens then were obtained from hospital pathology departments when possible. These methods have been described in detail elsewhere [29,30]. Briefly, >70% of the 1,310 cases and 5,273 controls who originally were identified for the study confirmed the diagnosis and granted permission, and specimens subsequently were obtained for 52% of those who had granted their permission (465 cases and 1,939 controls). The primary reason given by hospital pathology departments for not sending specimens was that they had been destroyed or were no longer available (35%) [29,30]. Biopsy slides were independently reviewed by one of two study pathologists (SJS, JLC) who were blinded to the participants' case or control status. The pathologists completed a detailed worksheet with information on the morphologic features of each specimen, and biopsies were then classified as nonproliferative, proliferative without atypia, or AH, according to the criteria of Page *et al. *[31,32], which have been used in previous investigations in this cohort [29,33,34]. All biopsies, including bilateral biopsies, were classified according to the most severe changes present, and specimens with possible or definite AH were jointly reviewed by both pathologists. After excluding participants whose benign biopsy specimens were of poor quality or had no breast tissue, evidence of carcinoma *in situ *or invasive carcinoma, invalid dates of diagnosis, or insufficient information on laterality, there were a total of 395 cases and 1,610 controls [29,30]. Although we were successful in obtaining slides for only 30% of those originally identified, the success rate did not differ significantly between those with a subsequent breast cancer diagnosis and those without. In addition, the breast cancer risk factors for women from whom we were successful in obtaining pathology specimens were very similar to those for the women for whom we were unable to obtain specimens, suggesting that these women were missing at random.

During histologic review of benign breast biopsies for breast cancer cases reported in the 1976 through 1996 questionnaires in the NHS and in the 1991 through 1995 questionnaires in the NHS II and for matched controls, the presence of CCL was recorded on the pathology worksheets as "columnar alteration of lobules." At the time these benign breast biopsies were reviewed, CCL were not considered to be of particular interest and were usually not further stratified; thus, the categorization of these lesions as "columnar alteration of lobules" was in keeping with the standard of practice of the time. For the 42 cases and 116 controls from the NHS 1996 questionnaire cycle, presence of CCL subtypes (columnar cell change, columnar hyperplasia, and flat epithelial atypia) was assessed rather than the overall presence of CCL. These women with only subtype information were re-categorized by presence or absence of CCL to allow their inclusion in analyses. After exclusion of five subjects having lesions without adjacent tissue for evaluation, the final sample for this analysis included 394 cases and 1,606 controls. Given the small number of cases and controls in which CCL were further classified with regard to the presence or absence of atypia, CCL were considered as a single group for these analyses.

### Statistical analysis

The distributions of breast cancer risk factors were examined according to CCL presence among the controls, adjusting for age at benign biopsy when appropriate. Unconditional logistic regression was used to estimate odds ratios (ORs) and 95% confidence intervals (95% CIs) for breast cancer risk according to CCL presence. Unconditional rather than conditional logistic regression was used because this analytic approach allowed us to use all cases and controls for whom we had histologic information. We first adjusted only for the matching factors, which were age at breast cancer diagnosis or index date (<45 years, 45 to 49 years, 50 to 54 years, 55 to 59 years, ≥60 years), year of benign breast biopsy (before 1970, 1970 to 1979, 1980 to 1989, 1990 and later), and time since benign biopsy (years from BBD diagnosis to the breast cancer diagnosis or index date, as a continuous variable). In another set of models, additional adjustment was made for histologic category of BBD (nonproliferative, proliferative without atypia, or AH), which is a strong predictor of breast cancer risk [31]. We also jointly classified women according to histologic category and CCL presence to examine whether the association of CCL with breast cancer risk differed by category of BBD; a likelihood ratio test was used to test for interaction.

The following covariates were considered as potential confounding factors: first-degree family history of breast cancer (yes, no), age at menarche (<12 years, 12 years, 13 years, ≥14 years), menopausal status (premenopausal, postmenopausal, dubious/unsure), jointly classified parity and age at first birth (nulliparous, 1 to 2 children and <25 years, 1 to 2 children and 25 to 29 years, 1 to 2 children and ≥30 years, ≥3 children and <25 years, ≥3 children and ≥25 years), BMI at age 18 (<21, 21 to 22.9, 23 to 24.9, 25+ kg/m^2^), postmenopausal hormone use (premenopausal, postmenopausal never, ever), and oral contraceptive use (never, ever). Because BMI at age 18 appeared to be more strongly related to breast cancer risk than current BMI, we chose to consider adjustment for BMI at age 18 and change in weight since age 18 rather than current BMI. Individual adjustment for these covariates did not change the estimated OR substantially, nor did simultaneous adjustment for the four factors having the strongest influence on the OR (BMI at age 18, family history of breast cancer, postmenopausal hormone use, and jointly classified parity/age at first birth). We examined relationships between CCL and estrogen receptor (ER) status, grade, and ipsilaterality in additional unconditional logistic regression models. All statistical analyses were performed using the SAS software package (version 9.1; SAS Institute, Cary, NC, USA). All tests were two-sided, with *P *< 0.05 indicating statistical significance.

## Results

The mean age at benign breast biopsy among all participants was 43.7 years (SD = 10.0 years), and the mean time since biopsy was 10.0 years (SD = 7.2 years). Compared with women with no CCL, women with CCL were older at the time of their biopsy and had less time since their biopsies (Table 1). Women with and without CCL were generally similar with regard to their distribution of breast cancer risk factors, although women with CCL were slightly leaner than those without CCL and were less likely to have used postmenopausal hormones. Histologic subtype of BBD was strongly associated with presence of CCL, with women having CCL much more likely to have proliferative disease and AH compared with women without CCL (*P *< 0.0001). We also assessed breast cancer risk factors by case-control status and found that established breast cancer risk factors were associated with breast cancer in our study population, although most of these associations were non-significant. Compared with controls, cases had a higher prevalence of family history of breast cancer, lower mean parity and older mean age at first birth among parous women, higher mean alcohol consumption, and a higher prevalence of postmenopausal hormone use (see Supplementary table S1 in Additional file 1).

**Table 1:** Characteristics by CCL status among controls with BBD*

	CCL	No CCL
No. of controls (%)	448 (27.9)	1,158 (72.1)
**Means**		
Age at BBD biopsy, years	45.1	43.3
Year of BBD biopsy (median)	1979	1979
Time since BBD biopsy, years	9.3	10.1
No. of BBD biopsy slides†	5.7	5.5
Body mass index, kg/m^2^†	24.2	25.0
Body mass index at age 18, kg/m^2^†	20.5	21.1
Weight gain after age 18, kg†	10.0	10.7
Age at menarche, years†	12.7	12.6
Age at first birth, years (parous only)†	24.9	24.9
Alcohol intake, g/day†	4.2	4.0
Parity (parous only)†	3.0	3.0
**Percentages**		
Premenopausal†	36.0	35.5
Parous†	91.9	92.4
First degree family history of breast cancer†	19.0	17.6
Ever postmenopausal hormone use†	32.0	37.3
Ever oral contraceptive use†	45.1	49.6
Histologic category of BBD†		
Nonproliferative	21.5	44.4
Proliferative without atypia	61.8	48.4
Atypical hyperplasia	16.8	7.2

CCL indicates columnar cell lesions; BBD, benign breast disease.

*Unless otherwise specified, all variables correspond to the time period immediately prior to the index date.

†Means and percentages were standardized to the age distribution of the controls at the time of benign biopsy, in five-year categories.

In the multivariate logistic regression models controlling for matching factors only (Table 2), women with CCL had a significantly higher risk of breast cancer compared with those without CCL (OR = 1.44; 95% CI, 1.14 to 1.83). However, after additional adjustment for histologic category of benign breast disease, this association became non-significant (OR = 1.20; 95% CI, 0.94 to 1.54). These associations were very similar when only invasive breast cancer cases were included (OR = 1.44; 95% CI, 1.11 to 1.86 with adjustment for matching factors only and OR = 1.18; 95% CI, 0.90 to 1.55 with additional adjustment for histologic category of BBD).

**Table 2:** ORs and 95% CIs for CCL and breast cancer risk among participants with BBD

	Cases	Controls	OR (95%CI)*	OR (95%CI)**
**CCL status**				
No CCL	254	1,158	1.0 (reference)	1.0 (reference)
CCL	140	448	1.44 (1.14, 1.83)	1.37 (1.06, 1.76)
**CCL status, adjusted for BBD histologic category**				
No CCL	254	1,158	1.0 (reference)	1.0 (reference)
CCL	140	448	1.20 (0.94, 1.54)	1.16 (0.89, 1.50)
**Jointly classified BBD/CCL histologic category**				
Nonproliferative, no CCL	80	518	1.0 (reference)	1.0 (reference)
Nonproliferative, CCL	19	93	1.33 (0.77, 2.29)	1.24 (0.69, 2.22)
Proliferative without atypia, no CCL	125	558	1.46 (1.08, 1.99)	1.38 (1.00, 1.91)
Proliferative without atypia, CCL	74	277	1.81 (1.27, 2.58)	1.63 (1.12, 2.38)
Atypical hyperplasia, no CCL	49	82	4.26 (2.75, 6.59)	4.04 (2.57, 6.36)
Atypical hyperplasia, CCL	47	78	4.37 (2.81, 6.81)	4.20 (2.63, 6.70)

OR indicates odds ratio; 95% CI, 95% confidence interval.

*Adjusted for age, year of BBD diagnosis, and time since BBD biopsy.

**Adjusted for the above-mentioned factors and body mass index at age 18, weight change between age 18 and diagnosis/index date, family history of breast cancer, postmenopausal hormone use, and jointly classified parity/age at first birth.

When women were jointly classified according to histologic category and CCL presence, all groups had an elevated breast cancer risk compared with the reference category, nonproliferative BBD and no CCL. Women with both AH and CCL were at highest risk (OR = 4.37; 95% CI, 2.81 to 6.81), but this risk was not significantly different from that among those with AH without CCL. Within all BBD histologic categories, breast cancer risk was elevated for women with CCL, with the risk higher for those with less severe BBD (nonproliferative BBD: OR = 1.36, 95% CI: 0.79 to 2.37; proliferative BBD without atypia: OR = 1.21, 95% CI: 0.87 to 1.68; and AH: OR = 1.10, 95% CI: 0.65 to 1.87; adjustment for matching factors only). However, there was no evidence of an interaction between CCL and BBD histologic category when either three histologic categories were used (*P *= 0.77) or when proliferative disease without atypia and AH were collapsed into a single category (*P *= 0.93).

There was some suggestion that women with CCL had a higher risk of ER positive and ipsilateral breast cancer compared with women without CCL, but these associations were not significant after adjustment for BBD histologic category. Breast cancer grade appeared to be unrelated to CCL status.

## Discussion

Our results in this established nested-case control study of BBD point to a modestly increased risk of breast cancer among women with CCL. However, it appeared that most of this elevation in risk was due to the close association between CCL and proliferative disease, which itself is an established risk factor for subsequent breast cancer [31]. Despite the apparent lack of an independent association between CCL and breast cancer, there was an indication that CCL may influence breast cancer risk among women with nonproliferative disease.

Few previous epidemiologic analyses have evaluated the risk of subsequent breast cancer following detection of CCL, but the magnitude of risk associated with the presence of CCL found in our study is consistent with that reported by Boulos *et al. *in a large retrospective cohort study among women in the Nashville Breast Cohort. Similar to our study, the Nashville study found a nearly 50% elevation in breast cancer risk associated with CCL (RR = 1.47; 95% CI: 1.0 to 2.2), although this analysis excluded those with AH, who have been shown to be at highest risk of subsequent breast cancer. Given the strong correlation between CCL and concurrent AH found by Boulos *et al. *(*P *< 0.0005), it is possible that the relative risk for presence of CCL would have been somewhat higher had women with AH been included in the main analysis. As in our study, Boulos *et al. *found that the elevated risk of breast cancer among those with AH was not further increased among those who additionally had CCL, suggesting that CCL are not an independent breast cancer risk factor among women with AH [25]. Another recent analysis by Kabat *et al. *that assessed subsequent breast cancer risk from a broad range of benign breast lesions found a non-significantly elevated risk of breast cancer among a small number of women with multiple CCL compared with those with non-proliferative disease and no breast pathology (RR = 1.72, 95% CI: 0.87 to 3.41), although no increased risk was found for focal CCL (RR = 0.81, 95% CI: 0.47 to 1.39) [27]. However, it is difficult to compare our results with those from the study by Kabat *et al. *because this study used a different pathologic classification scheme in which CCL were considered a proliferative disease rather than a separate entity that could exist in conjunction with either nonproliferative or proliferative BBD.

Our study is the largest nested case-control study of CCL and breast cancer to date and therefore yields important clues into the relationship between presence of CCL and breast cancer risk. However, given the heterogeneity of CCL, it is possible that assessing risk of breast cancer for all CCL subtypes combined masks an effect of advanced CCL subtypes, which are less common. Histopathologic and molecular evidence suggests that CCL displaying low-grade cytologic atypia, recently termed "flat epithelial atypia" (FEA) by the World Health Organization Working Group on the Pathology and Genetics of Tumors of the Breast [35], may be a direct precursor to low-grade DCIS, while the more common non-atypical CCL are not thought to be precancerous [36].

However, the malignant potential of FEA remains equivocal, as descriptive and epidemiologic studies fail to support molecular findings, just as they do for overall CCL risk. Most descriptive follow-up studies among women with what would now be classified as FEA, including one with an average follow-up of nearly 20 years, have found progression to invasive carcinoma to be uncommon [6,11,23,24]. Three previous observational studies have assessed CCL subtype-specific risks, but all had limited power to detect associations for FEA due to small numbers of women with this subtype. Two of these studies did find an increased risk among women with atypical CCL compared with those having nonproliferative disease [27] or with all women without atypical CCL [26], but results were non-significant and confidence intervals were very wide, as one study had 19 women with atypical CCL [26] and the other had only 6 [27]. The largest of the three studies found no evidence of an increased breast cancer risk associated with "columnar cell lesions with atypia" among women without AH. This study did indicate that risk may be increased for those having CCL with hyperplasia, although this could have been a chance finding due to the small number of women with each CCL subtype even in this larger analysis [25].

Although we considered performing a CCL subtype-specific analysis, information on subtype (columnar cell change, columnar hyperplasia, and FEA) was collected for only 157 women whose breast cancer or index date was reported on the 1996 NHS questionnaire. We chose not to re-review the biopsy slides for the presence of FEA, as prevalence of FEA appeared very low from the 1996 questionnaire cycle, with only 4 of the women out of 53 with CCL from this cycle having FEA. Because FEA is a rare occurrence, many thousands of breast biopsies would be needed for adequate power to study its relationship with subsequent breast cancer. Moreover, to study the association between FEA and breast cancer independent of AH, an adequate number of women with FEA but not AH would be required. This would only be possible in an extremely large study, as FEA commonly occurs in conjunction with AH. Even in the largest previous epidemiologic study of CCL by Boulos *et al.*, only 52 biopsies contained FEA without AH [25].

The strengths of the study include its prospective design, the confirmation of breast cancer cases through review of medical records, the centralized pathology review of specimens from benign biopsies before the development of breast cancer, and the detailed information on other breast cancer risk factors. Furthermore, ours is the first observational study to compare the risk of subsequent breast cancer by BBD histologic category. Although power was limited to test for heterogeneity by histologic category, our findings suggest that CCL may be a marker of increased risk among those with non-proliferative disease. Such a differential effect of CCL on breast cancer risk across BBD categories could have important implications for clinical management of patients with CCL, especially given that women with non-proliferative disease are typically considered to be at no elevated risk for breast cancer compared with women from the general population.

## Conclusions

In light of these findings, large epidemiologic studies assessing CCL subtype-specific risks are needed to inform clinical guidelines for the management of CCL. In particular, better understanding is needed as to whether CCL with atypia in the absence of more advanced lesions require treatment similar to that for DCIS. Additionally, adequately powered epidemiologic studies might help elucidate whether CCL are of concern only among women without proliferative disease. Regardless of whether particular CCL subtypes are simply markers for premalignant changes in the breast or precancerous histologic changes themselves, recognition of these lesions is nevertheless important, as detection of CCL via biopsy should prompt additional scrutiny for other pathologic changes in breast tissue that may signal the early development of DCIS or invasive carcinoma.

## Abbreviations

AH: atypical hyperplasia; BBD: benign breast disease; BMI: body mass index; CCL: columnar cell lesions; CI: confidence interval; DCIS: ductal carcinoma *in situ*; ER: estrogen receptor; FEA: flat epithelial atypia; NHS: Nurses' Health Study; OR: odds ratio.

## Competing interests

The authors declare that they have no competing interests.

## Authors' contributions

SA participated in the study design, data analysis and interpretation, and manuscript drafting. LC participated in the study design, data collection, and manuscript revision. SS and JC reviewed the BBD biopsy slides and participated in the study design and manuscript revision. GC participated in the study design, data collection, and manuscript revision. RT participated in study design, data collection, manuscript revision, and study supervision. All authors read and approved the final manuscript.

## Supplementary Material

Additional file 1 **Supplementary table S1**. A Word document containing a table that lists the means and percentages of characteristics by case-control status among participants with benign breast disease (BBD). Click here for file
