# Supplementary material for: Columnar cell lesions and subsequent breast cancer risk: a nested case-control study
Source: Breast Cancer Res. 2010 Aug 6;12(4):R61. doi: 10.1186/bcr2624 (PMC2949654; doi:10.1186/bcr2624)
Supplement: Additional file 1 — Supplementary table S1. A Word document containing a table that lists the means and percentages of characteristics by case-control status among participants with benign breast disease (BBD). [file bcr2624-S1.DOCX]

| **Supplementary Table 1. Characteristics by Case-Control Status among Participants with BBD*** | | | |
| --- | --- | --- | --- |
|  | **Cases** | | **Controls P-value** |
|  | |  |  |
| No. of participants (%) | | 394 | 1606 |
|  | |  |  |
| **Means** | |  |  |
| Body mass index, kg/m^2^ | | 24.5 | 24.8 0.23 |
| Body mass index at age 18, kg/m^2^ | | 20.5 | 20.9 0.0063 |
| Age at menarche, years | | 12.5 | 12.6 0.21 |
| Age at first birth, years (parous only) | | 25.1 | 24.9 0.20 |
| Alcohol intake, g/day | | 4.2 | 4.1 0.78 |
| Parity (parous only) | | 2.9 | 3.0 0.20 |
|  | |  |  |
| **Percentages** | |  |  |
| Premenopausal | | 36.0 | 35.9 0.97 |
| Parous | | 92.6 | 92.3 0.84 |
| First degree family history of breast cancer | | 25.1 | 17.9 0.0013 |
| Ever postmenopausal hormone use | | 36.8 | 35.5 0.64 |
| Ever oral contraceptive use | | 49.5 | 48.4 0.69 |
| Histologic category of BBD | |  |  |
| Nonproliferative | | 25.1 | 38.0 <0.0001 |
| Proliferative without atypia | | 50.5 | 52.0 0.60 |
| Atypical hyperplasia | | 24.4 | 10.0 <0.0001 |

BBD indicates benign breast disease. *Unless otherwise specified, all variables correspond to the time period immediately prior to the diagnosis or index date.
